# Comprehensive clinical profiling of the Gauting locoregional lung adenocarcinoma donors

**DOI:** 10.1002/cam4.2031

**Published:** 2019-02-25

**Authors:** Laura V. Klotz, Yves Courty, Michael Lindner, Agnès Petit‐Courty, Anja Stowasser, Ina Koch, Martin E. Eichhorn, Ioannis Lilis, Alicia Morresi‐Hauf, Kristina A. M. Arendt, Mario Pepe, Ioanna Giopanou, Giannoula Ntaliarda, Sabine J. Behrend, Maria Oplopoiou, Valérie Gissot, Serge Guyetant, Sylvain Marchand‐Adam, Jürgen Behr, Jan‐Christian Kaiser, Rudolf A. Hatz, Anne‐Sophie Lamort, Georgios T. Stathopoulos

**Affiliations:** ^1^ Center for Thoracic Surgery Munich Ludwig‐Maximilians‐University of Munich (LMU) and Asklepios Medical Center Member of the German Center for Lung Research (DZL) Gauting Bavaria Germany; ^2^ Comprehensive Pneumology Center and Institute for Lung Biology and Disease University Hospital Ludwig‐Maximilians University of Munich (LMU) and Helmholtz Center Munich Member of the German Center for Lung Research (DZL) Munich Bavaria Germany; ^3^ French National Institute of Health and Medical Research (INSERM) Unit 1100 Faculty of Medicine Research Center for Respiratory Diseases (CEPR) University F. Rabelais Tours Cedex Centre France; ^4^ Department of Thoracic Surgery Ruprecht‐Karls‐University of Heidelberg Heidelberg Baden‐Württemberg Germany; ^5^ Laboratory for Molecular Respiratory Carcinogenesis Department of Physiology Faculty of Medicine University of Patras Biomedical Sciences Research Center Achaia Greece; ^6^ Department of Pathology Asklepios Medical Center Gauting Bavaria Germany; ^7^ INSERM Center for Clinical Investigation (CIC) Unit 1415 Regional University Hospital Center (CHRU) Tours Bretonneau Hospital Tours Cedex Centre France; ^8^ Regional University Hospital Center (CHRU) Tours Department of Pathology and Tumor Biobank Bretonneau Hospital Tours Cedex Centre France; ^9^ Department of Pneumology Asklepios Lung Clinic Gauting Member of the German Center for Lung Research (DZL) Gauting Bavaria Germany; ^10^ Institute of Radiation Protection (ISS) Helmholtz Center Munich Neuherberg Bavaria Germany

**Keywords:** LADC, lung adenocarcinoma, obstruction, smoking, survival

## Abstract

A comprehensive characterization of lung adenocarcinoma (LADC) clinical features is currently missing. We prospectively evaluated Caucasian patients with early‐stage LADC. Patients with LADC diagnosed between 2011 and 2015 were prospectively assessed for lung resection with curative intent. Fifty clinical, pathologic, radiologic, and molecular variables were recorded. Patients were followed till death/study conclusion. The main findings were compared to a separate cohort from France. Of 1943 patients evaluated, 366 were enrolled (18.8%; 181 female; 75 never‐smokers; 28% of registered Bavarian cases over the study period). Smoking and obstruction were significantly more prevalent in GLAD compared with adult Bavarians (*P* < 0.0001). Ever‐smoker tumors were preferentially localized to the upper lobes. We observed 120 relapses and 74 deaths over 704 cumulative follow‐up years. Median overall and disease‐free survival were >7.5 and 3.6 years, respectively. Patients aged <45 or >65 years, resected >60 days postdiagnosis, with abnormal FVC/DL_CO_V_A_, N2/N3 stage, or solid histology had significantly decreased survival estimates. These were fit into a weighted locoregional LADC death risk score that outperformed pTNM7 in predicting survival in the GLAD and in our second cohort. We define the clinical gestalt of locoregional LADC and provide a new clinical tool to predict survival, findings that may aid future management and research design.

## 1 INTRODUCTION

Lung adenocarcinoma (LADC) is the most frequent histologic type of lung cancer.^1^, ^2^ It constitutes the most deadly human cancer, causing 650 000 deaths per year worldwide,^3^, ^4^ while its incidence is increasing in active smokers, ex‐smokers, and never‐smokers.^5^ Simultaneously, LADC is the most frequent lung cancer in never‐smokers, women, and young patients, rendering understanding and treating the disease imperative.^5^, ^6^ LADC is mainly caused by smoking, radiation, and other exposures.^5^, ^7^ Although multiple approaches to prevention/early detection have been evaluated, only 15% of patients diagnosed with LADC are amenable to surgery, the only definitive cure.^8^ These patients are of tremendous importance, since they donate tissues for research that has fostered our understanding of the pathobiology of locoregional LADC and has enabled targeted therapies for patients with defined oncogenic driver mutations.^9^, ^10^

Recent molecular evidence indicates LADC to be a distinct disease entity.^11^ However, the clinical gestalt of the disease has not been comprehensively characterized separately from other forms of lung cancer. Here we report the first results from the Gauting locoregional lung adenocarcinoma donors (GLAD) study, a prospective biobank of LADC tissues and clinical phenotypes. The wealth of clinical information provided includes multiple variables and prolonged follow‐up data, enabling the discovery of new associations reported here, as well as the future establishment of genotype‐phenotype links.

## 2 MATERIAL AND METHODS

### 2.1 Studies approval

GLAD was conducted in accord with the Helsinki Declaration, reported in accord to STROBE (https://www.strobe-statement.org/index.php?xml:id=strobe-home), approved by the LMU Ethics Committee (623‐15), registered with the German Clinical Trials Register (http://www.drks.de/drks_web/navigate.do?navigationId=trial.HTML&TRIAL_ID=DRKS00012649), and written informed consent was obtained from all patients (https://www.asklepios.com/gauting/experten/experten/biobank/). The Tours study was conducted according to the Helsinki Declaration, was approved by the Ethics Committee of Région Centre (2015/051), and was registered with the French Ministry of Health (DC‐2008‐308). All patients gave written informed consent.

### 2.2 GLAD study

All patients with histologic LADC diagnosis at Asklepios Medical Center between February 2011 and September 2015 were prospectively evaluated for lung resection with curative intent. LADC was staged according to the current Seventh Edition of the International Association for the Study of Lung Cancer (IASLC) tumor–node–metastasis staging system (TNM7).^2^ Preoperative lung function was assessed according to current guidelines.^12^ The Absolute and percentage predicted values for forced vital capacity (FVC), forced expiratory volume in 1 sec (FEV_1_), FEV_1_/FVC ratio, lung diffusion capacity for carbon monoxide (DL_CO_), and DL_CO_ corrected for alveolar ventilation (DL_CO_/*V*_A_) were recorded. Patients eligible and fit for surgery were prospectively enrolled. Baseline data obtained at entry were: blinded patient identifier (ID), age and sex, body mass and length, date and mode of clinical and tissue diagnosis, clinical TNM7 (cTNM7) stage including site and extent of metastatic disease, smoking start, stop, and intensity, and lung function results. Chronic obstructive pulmonary disease (COPD) was defined as smoking >30 pack‐years with compatible symptoms and FEV_1_/FVC <70% and was graded by the global initiative for chronic obstructive lung disease (GOLD) 2001 classification.^13^ All patients were re‐evaluated at 30 days postsurgery, the benchmark of referral to oncology/radiotherapy (all stage III/IV patients received adjuvant therapy) or dismissal to out‐patient follow‐up according to current guidelines.^14^ Data prospectively recorded included: date of surgery, time from diagnosis to treatment calculated from imaging/tissue diagnosis (whichever occurred first) to resection date, blinded tissue ID, lobar tumor location, relapse/metastasis date and site, histologic subtype, pathologic TNM7 (pTNM7) stage, and oncogene testing results. Follow‐up data were retrospectively acquired from visits, medical charts, telephone consultations with treating physicians, and/or death certificate searches and included: adjuvant therapy, relapse/metastasis date, site, and extent, and death or last contact. Primary endpoint was overall survival (OS), calculated from surgery to death (event) or last contact (censored); secondary endpoint was disease‐free survival (DFS), calculated from surgery until recurrence (event) or last contact (censored); tertiary endpoints were associations between the variables obtained.

### 2.3 Tours comparison cohort

All patients with tissue‐diagnosed LADC between January 2006 and December 2011 were prospectively evaluated for curative resection, staged according to TNM7,^2^ preoperatively tested for lung function, prospectively enrolled if eligible, and fit for surgery. Data obtained and endpoints were identical to GLAD, except from histologic subtype, extent of metastatic disease, and oncogene test results.

### 2.4 Histology and genotyping

LADC subtypes of GLAD were determined by our pathology expert (AMH) according to IASLC guidelines.^1^, ^2^

### 2.5 Statistics

Minimal study size (*n* ^MIN^) was determined by power analyses (http://www.gpower.hhu.de/en.html) employing Fisher's exact test, proportion inequalities in two independent groups, α error=0.05, 80% power, and 1:1 allocation ratio. *n* ^MIN^ = 314 was required to detect the difference between 0% and 5% and *n* ^MIN^ = 348 between 30% and 45%. We targeted recruitment to *n *=* *350 and achieved *n *=* *366 in September 2015. Data distribution was tested using Kolmogorov‐Smirnov test and summaries are given as frequencies or point estimates (mean or median) with descriptors of dispersion (standard deviation, SD or interquartile range, IQR or 95% confidence interval, 95%CI), as appropriate and indicated. Survival was analyzed by Kaplan‐Meier estimates and Cox proportional hazard models using Waldman backward elimination. Log rank tests were used for comparisons. Associations between variables were examined using Fisher's exact or *χ* ^2^ tests, Student's *t*‐ or Mann‐Whitney *U*‐tests, one‐way analysis of variance (ANOVA) with Bonferroni posttests or Kruskal‐Wallis ANOVA with Dunn's posttests, Pearson's or Spearman's correlations, and linear regression, depending on input and target variable nature and distribution, as appropriate and as indicated. Probabilities (*P*) < 0.05 were considered significant. Least absolute shrinkage and selection operator (LASSO) regression analysis was carried out using the GLMNET package on R*, where the number of regression coefficients shrunk according to a penalization factor λ (https://www.r-project.org/) and their point estimates were determined with cross‐validation using 244 samples with complete records. Unsupervised clustering of 362 GLAD patients was done using ConsensusCluster;^15^ settings were *K* = 2‐6, subsample size = 300, and fraction = 0.8, *K*‐means algorithm with average linkages, hierarchical consensus, and Euclidean distance metric, and center principal component analysis normalization with fraction = 0.85 and eigenvalue weight = 0.25. Receiver‐operator curves (ROC) were used to identify variables defining patient clusters. Analyses were done on the Statistical Package for the Social Sciences v24.0 (IBM, Armonk, NY) and Prism v5.0 (GraphPad, San Diego, CA).

## 3 RESULTS

### 3.1 The Gauting locoregional lung adenocarcinoma donors (GLAD)

**Figure 1:**
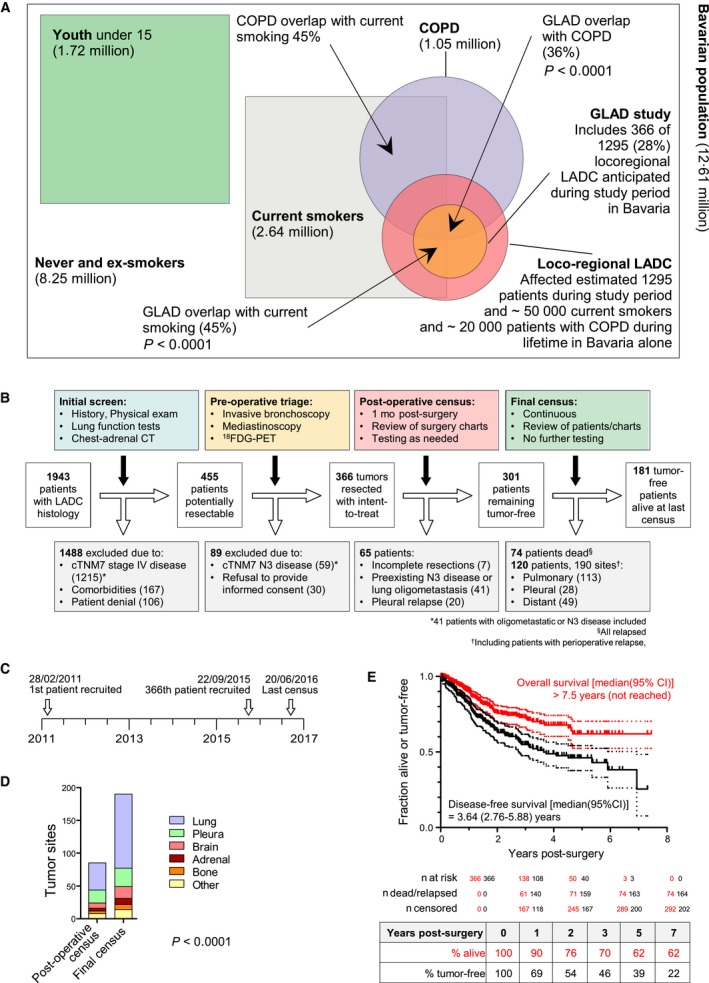
The Gauting locoregional lung adenocarcinoma donors (GLAD) study overview and main results at the mid‐2016 census. (A) Venn diagram of current smoking and COPD prevalence, and LADC incidence over the GLAD study period in Bavaria. Data were obtained from the present study, from the Bavaria cancer registry, and from references 16‐18. (B) Study flowchart. (C) Study timeline. (D) Cumulative relapse events observed by site at the 30‐day postresection and long‐term follow‐up benchmarks. Shown are number of observations (*n*) and *χ* ^2^ test probability (*P*). (E) Kaplan‐Meier plots and estimates of overall and disease‐free survival with patient numbers at risk, events observed, and patients censored (graph) and actual (excluding censored observations) percentage of patients surviving at 1, 2, 3, 5, and 7 years postresection (table)

**Figure 2:**
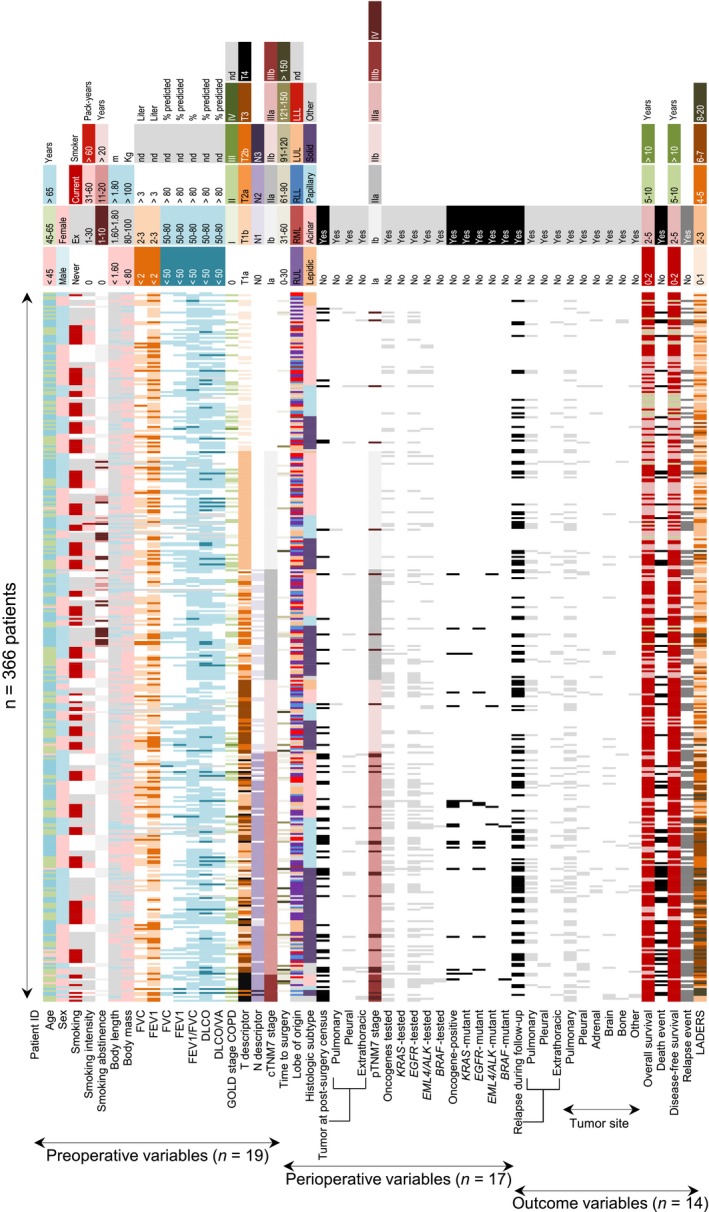
GLAD phenome plot. Color‐coded pivot table of all data obtained from GLAD sorted sequentially by cTNM7 stage, histologic subtype, sex, and smoking status. Columns represent individual patients and rows variables recorded at study entry, postsurgery census, and longitudinal follow‐up. The raw data table is provided as Table S2. *n*, sample size; ID, identifier; FVC, forced vital capacity; FEV_1_, forced expiratory volume in 1 seconds; DL_CO_, uncorrected lung diffusion capacity for carbon monoxide; *V*_A_, alveolar ventilation; COPD, chronic obstructive pulmonary disease; GOLD, global initiative for chronic obstructive lung disease; TNM, tumor‐node‐metastasis staging system; c, clinical; p, pathologic; RUL, right upper lobe; RML, right middle lobe; RLL, right lower lobe; LUL, left upper lobe; LLL left lower lobe; *EGFR*, epidermal growth factor receptor; *KRAS*, V‐Ki‐ras2 Kirsten rat sarcoma viral oncogene homolog; *BRAF,* v‐Raf murine sarcoma viral oncogene homolog B; *EML4*, echinoderm microtubule associated protein like 4; *ALK*, anaplastic lymphoma kinase; LADERS, locoregional lung adenocarcinoma death risk score; nd, not determined

**Figure 3:**
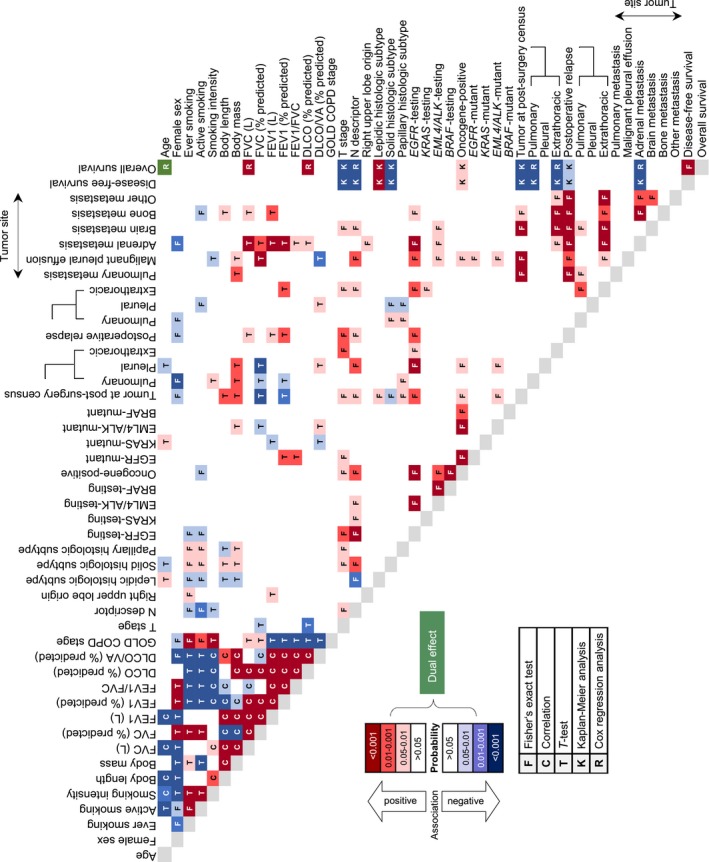
GLAD association heatmap. Color‐coded pivot table of all associations observed in the GLAD cohort. Colors represent the direction and probability of observed associations and letters the statistical method employed to detect them. *n*, sample size; FVC, forced vital capacity; FEV_1_, forced expiratory volume in 1 seconds; DL_CO_, uncorrected lung diffusion capacity for carbon monoxide; V_A_, alveolar ventilation; COPD, chronic obstructive pulmonary disease; GOLD, global initiative for chronic obstructive lung disease; TNM, tumor‐node‐metastasis staging system; c, clinical; p, pathologic; *EGFR*, epidermal growth factor receptor; *KRAS*, V‐Ki‐ras2 Kirsten rat sarcoma viral oncogene homolog; *BRAF*, v‐Raf murine sarcoma viral oncogene homolog B; *EML4*, echinoderm microtubule associated protein like 4; *ALK*, anaplastic lymphoma kinase

During the period from February 2011 to September 2015, 1943 patients with LADC were prospectively assessed in the Asklepios Medical Center, Gauting, Germany. Among them, 455 were eligible and fit for curative surgery, and 366 were enrolled (89 patients were excluded due to cTNM7 N3 disease or unwillingness to provide informed consent). They represent ~28% of registered Bavarian locoregional LADC cases during the study period (21 588 lung cancer cases, corresponding to 8635 LADC cases at expected 40%, and to 1295 resectable LADC cases at expected 15%; http://www.krebsregister-bayern.de/index_e.html) summarized in Figure 1A.^16^, ^17^, ^18^ During the same period, another 1577 patients with LADC were not eligible or fit for lung resection, rendering 23% of patients screened resectable with intention to treat, and resulting in 19% recruitment rate into GLAD (Figure 1B and C). Of the 366 patients resected, 41 had oligometastatic disease detected prior to surgery, seven were incompletely resected, and in 20 a malignant pleural disease was identified intraoperatively. Out of the 305 patients that were tumor‐free after surgery, 301 remained tumor‐free at the 30‐day postoperative census (82.2%), and 181 (49.5%) at the mid‐2016 census (Figure 1D). At this time, 8453 cumulative follow‐up months (median[interquartile range, IQR] 18 [7‐33] months/patient) had been delivered, and 120 relapses and 74 deaths were observed. Median(95% confidence interval, CI) overall survival (OS) was not reached (>7.5 years), disease‐free survival (DFS) was 3.64 (2.76‐5.88) years, and 5‐year OS and DFS rates were 62% and 39%, respectively (Figure 1E). GLAD will be re‐censored mid‐biannually; hence survival data are expected to evolve. A color‐coded phenome plot of all information available at the mid‐2016 census is shown in Figure 2 and Table S1, while a heat map of all the associations observed (discussed below) is given in Figure 3. The major findings from GLAD classified according to clinical variables are presented below.

### 3.2 Age

**Figure 4:**
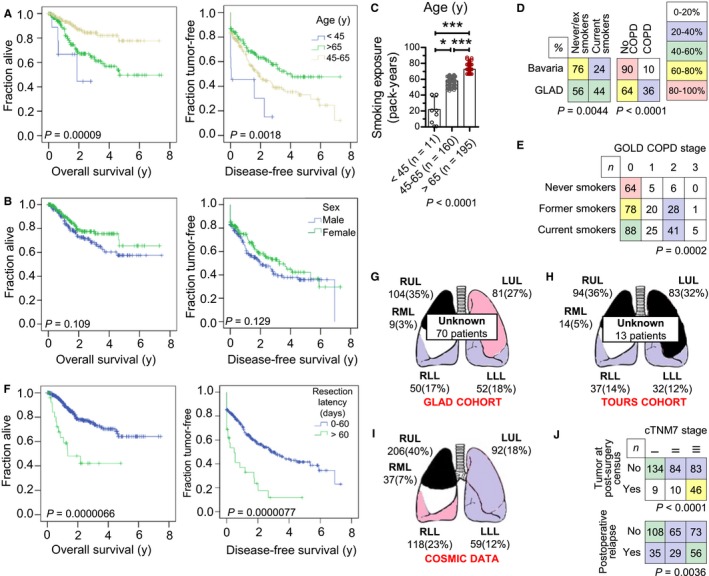
Incidence of different clinical parameters on LADC development in GLAD. (A, B, F) Kaplan‐Meier disease‐free and overall survival plots and overall log‐rank test probability values (*P*) of the GLAD. (A) Stratified by age (*n* = 11, 160, and 195, respectively, for age groups <45, 45‐65, and >65 years). (B) Stratified by sex (*n* = 181 and 185, respectively, for women and men). (C) Smoking exposure stratified by age. Shown are patient numbers (*n*), raw data points (dots), mean (columns), SD (bars), and Kruskal‐Wallis test probability (*P*). * and ***: *P* < 0.05 and *P* < 0.001, respectively, for the indicated comparisons by Dunn's posttests. (D) Crosstabulations of current smoking and COPD prevalence in GLAD and in Bavaria. Data were obtained from the present study and from references 22 and 24. COPD was staged according to the GOLD classification (28). Shown are percentages and Fisher's exact probability (*P*). (E) Crosstabulation of smoking status and GOLD COPD stage in GLAD. Shown are patient numbers (*n*) and *χ* ^2^ probability (*P*). (F) Stratified by timely (*n* = 337) or delayed (*n* = 29) resection and by complete (*n* = 310) or incomplete (*n* = 56) resection. (G‐H) LADC location by lung lobe determined at surgery (G) in the GLAD derivation cohort, (H) in a smoking‐optimal comparison cohort from Tours, France, (I) in COSMIC database (https://cancer.sanger.ac.uk/cosmic). Shown are schematic representations of the lungs with their lobes (RUL, right upper lobe; RML, right middle lobe; RLL, right lower lobe; LUL, left upper lobe; LLL, left lower lobe) and the number (*n*) and percentage of tumors observed. Color indicates frequency. (J) Crosstabulations of relapse events in the GLAD by cTNM7 stage. Shown are patient numbers (*n*), color‐coded frequencies by age grouping, and Fisher's exact probabilities (*P*)

In GLAD, median(IQR) age was 67 (59‐72) years, including 11 (3%) and 195 (53%) patients younger than 45 and older than 65 years, respectively; those had markedly decreased overall survival (OS) and disease‐free survival (DFS) compared with 160 (44%) patients aged between 45 and 65 years (Figure 4A and B). Age was positively associated with cumulative smoke exposure and lepidic/papillary histology. On the contrary, it was negatively linked with current smoking, body length, FVC and FEV_1_, and time to surgery (Figure 3). In addition, more death and relapse events were observed in patients of extreme age (<45 or >65 years) (Figure S1A). Linear regression‐calculated lung function decline rates with age were similar to the Framingham study,^19^ and lung function test results were tightly correlated with body metric indices, validating GLAD lung function data (Figure S1B‐D). Interestingly, patients with affected resection margins and perioperative pleural relapse were significantly younger (Figure S1A).

### 3.3 Sex

Surprisingly, 181 patients (49.5%) of GLAD were female, reflecting increasing local and worldwide female smoking trends.^6^, ^20^ Female sex was positively associated with percent predicted FVC and FEV_1_ values and FEV_1_/FVC ratio, and negatively linked with smoking rate and intensity, body metric indices, absolute FVC and FEV_1_ and percent predicted DL_CO_/*V*_A_, COPD frequency, solid histologic subtype, and adrenal relapse (Figure S2). However, sex did not significantly impact survival (Figure 4B). These results suggested that locoregional LADC in Caucasian women has distinct features as proposed elsewhere.^6^ However, these do not profoundly alter the biologic course of the disease, in accord with published results from Norway.^20^

### 3.4 Smoking

The GLAD study included 75 never (20.5%), 130 former (35.5%), and 161 current (44.0%) smokers. Alarmingly, active smokers were younger (Figure 4C). Smoking abstinence of ex‐smokers was median(IQR) = 10(5‐25) years. Importantly, more than 50% of patients were never/ex‐smokers (Figure 1A). GLAD smoking rates were disproportional to a Norwegian cohort of 54 never (7.8%), 255 former (36.8%), and 383 current (55.3%) smokers (*P *<* *0.0001 compared with GLAD, *χ* ^2^ test),^20^ but proportional to a French cohort from Tours that included 39 never (14.3%), 102 former (37.4%), 124 current (45.4%), and 8 indeterminate (2.9%) smokers (*P *=* *0.1114 compared with GLAD, *χ* ^2^ test).^21^ Hence the Tours cohort was identified as an optimal comparison set (Figure S3, Table S2). Expectedly, current smoking was significantly more frequent (*P *=* *0.0044, *χ* ^2^ test) in GLAD (44%) compared with current Bavarian rates (24.2%) (Figure 4D).^17^ Median(IQR) pack‐years smoked were 40 (9‐60), and smoking exposure correlated negatively with lung function, especially DL_CO_/*V*_A_ (Figure S4A‐C). Moreover, in accord with published results,^22^ active smoking was associated with solid, and never smoking with acinar histology (Figure S4D). Interestingly, smoking was negatively associated with N stage, postoperative pleural relapse, as well as bone metastasis (Figure S4D).^9^ However, smoking did not affect survival (Figure S4E and F). Collectively the data indicate that smoking is intimately linked with LADC and suggest that active smoking continuously drives the disease in the lungs, likely via tumor‐promoting effects of nicotine.^23^

### 3.5 Obstruction and COPD

When GLAD were classified according to original GOLD criteria,^13^ patients had stage 0 (62.8%), 50 patients stage I (13.7%), 75 patients stage II (20.5%), 6 patients stage III (1.6%), and 5 patients indeterminate (1.4%) COPD status (Figure 2). Smoking was intimately linked with GOLD COPD stage (*P *<* *0.0001, Fisher's exact test) and COPD was significantly more prevalent in GLAD compared with current Bavarian rates (*P *<* *0.0001, Fisher's exact test; Figure 4D and E).^18^ These findings were validated using real‐time statistics (https://knoema.com/REG_DEMO_TL2/demographic-statistics?region=1001010-bavaria, http://www.registrecancers59.fr/index.php/incidence) in GLAD and Tours cohorts (Figure 1A, Figure S3A),^24^ underpinning the causative role of smoking in both COPD and LADC.^25^ Lung function tests were concordant to GOLD COPD definition (Figure S5A). COPD was positively associated with affected resection margins and perioperative pleuropulmonary relapse likely attributable to adverse effects of distorted lung structure on surgical outcome, and correlated negatively with FVC and DL_CO_/*V*_A_ (Figure S5B). However, COPD did not impact survival (Figure S5C and D). Of all lung function variables, only abnormal percentage predicted FVC and DL_CO_/*V*_A_ negatively impacted survival (Figure S5E and F). Collectively, the data indicate that COPD and LADC show significant overlap, suggesting a common pathogenesis, in line with the literature.^25^ Moreover, the percentage predicted FVC and DL_CO_/*V*_A_, but not other spirometry indices or a diagnosis of COPD, can predict survival.

### 3.6 cTNM7 staging

All patients were staged according to cTNM7 to guide management.^2^, ^14^ We included history, physical exam, and chest‐to‐adrenal computed tomography in all. For stage III patients, an invasive bronchoscopy with mediastinal lymph node sampling, mediastinoscopy, and/or ^18^fluoro‐deoxyglucose positron emission tomography were also performed. Analysis of T, N, and cTNM7 stage showed a significant impact on survival (Figure S6) and validated GLAD against the reference IASLC study.^2^

### 3.7 Surgery

Time from imaging/tissue diagnosis to surgery was median(IQR) = 6 (0‐25) days. Resection within 60 days was achieved in 337 patients (92%), while 29 (8%) had resections performed >60 days after diagnosis. Out of 366 GLAD patients, 58 had preexisting oligometastatic, N3 disease, or pleural dissemination newly identified at surgery, leaving 308 for resection with intent‐to‐treat. Complete resection was achieved in 301 of these patients (97.7%; *P *=* *0.8639, Fisher's exact test). Importantly, time to surgery significantly affected overall and disease‐free survival (Figure 4F).

### 3.8 Tumor location

The lobe of origin of GLAD tumors was definitively determined during surgery in 296 patients, while tumors involving multiple lobes, central airways, and/or mediastinal structures rendered this impossible in 70 patients. We identified a striking upper lobe predominance in both GLAD and Tours cohorts, which was disproportional to published lobe ventilation or perfusion patterns, and was reminiscent of lobar ventilation/perfusion ratios (Figure 4G‐I).^26^, ^27^ Strikingly, RUL LADCs predominated in smokers of both cohorts, and patients with RUL LADC displayed higher FVC, FEV_1_, and N stage, but similar survival, compared with all other patients (Figure S7).

### 3.9 Histology

After the pathologic review of multiple tumor sections and sites (AMH), GLAD were classified into 16 lepidic (4.4%), 141 acinar (38.5%), 70 papillary (19.1%), 126 solid (34.4%), 2 fetal (0.5%), 2 adenosquamous (0.5%), 4 micropapillary (1.1%), and 5 indeterminate (1.4%) histologic subtypes. Papillary histology was more frequent compared with a published reference cohort that comprised 41 lepidic (8.2%), 207 acinar (41.4%), 23 papillary (4.6%), 183 solid (36.6%), 33 micropapillary (6.6%), and 13 indeterminate (2.6%) locoregional LADC (*P *<* *0.0001, *χ* ^2^ test).^22^ Encouragingly, indeterminate tumor rate was low in both studies, indicating the reproducibility of the IASLC classification.^1^, ^2^, ^23^ In accord with the above‐referenced study,^22^ lepidic‐predominant tumors in LADC was more frequent in never‐smokers and displayed lower overall TNM descriptors, decreased metastatic propensity, and prolonged overall survival, as opposed to solid‐predominant LADC that displayed aggressive features and poor survival (Figure S8), further validating GLAD.

### 3.10 Patterns of relapse

**Figure 5:**
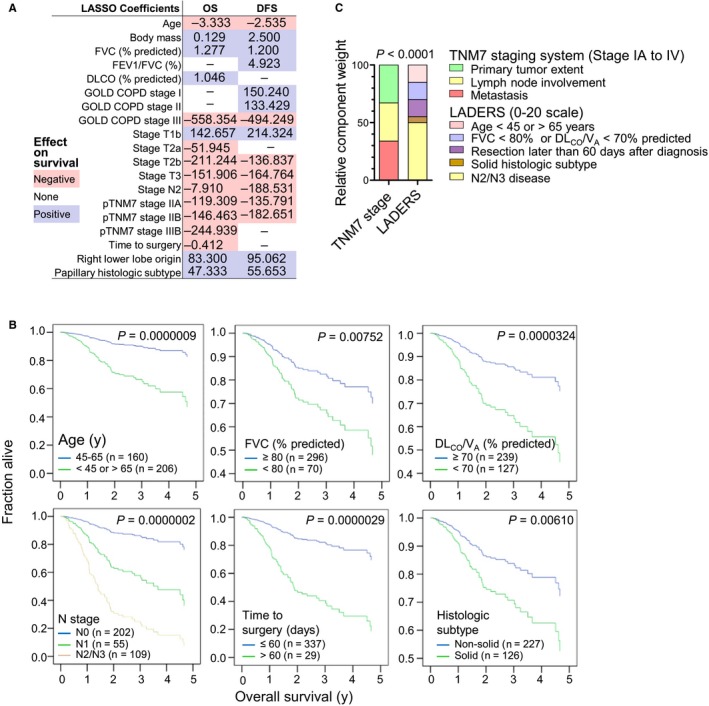
Development of the locoregional lung adenocarcinoma death risk score (LADERS) from the GLAD derivation cohort. (A) Results of least absolute shrinkage and selection operator (LASSO) regression. Shown are regression coefficients for overall (OS) and disease‐free (DFS) survival and color‐coded direction of impact on survival. (B) Results of Cox regression showing proportional hazards survival plots for the six independent predictors of survival of GLAD, including sample sizes (*n*) and probability values (*P*). (C) Schematic representation of the components and relative weight of the variables that comprise LADERS compared with the TNM staging system, including *χ* ^2^ probability value (*P*). FVC, forced vital capacity; FEV_1_, forced expiratory volume in 1 seconds; DL_CO_, uncorrected lung diffusion capacity for carbon monoxide; *V*_A_, alveolar ventilation; GOLD, global initiative for chronic obstructive lung disease; COPD, chronic obstructive pulmonary disease; TNM, tumor‐node‐metastasis staging system; p, pathologic

Over 704 cumulative follow‐up years, 190 relapse events were identified in 120 patients (Figure 1D). In addition to the associations described above, patients with higher cTNM7 descriptors had higher relapse rates, both at the 30‐day postsurgery and at mid‐2016 benchmarks (Figures 4J and 5). Relapse timing and site did not significantly impact OS; however, pleural or multi‐site relapse (5/20 patients with multiple relapses also had pleural relapse) adversely impacted DFS indicating that pleural relapse occurs earlier than others (Figure S9).

### 3.11 Survival

We next assessed the impact of each variable on OS and DFS. In a first step, Kaplan‐Meier analyses using OS and DFS as target and single variables as inputs (continuous numerical variables were dichotomized at abnormal cutoffs) showed that patients with age outlying 45‐65 years, abnormal percentage predicted FVC and DL_CO_/*V*_A_, high T, N, and cTNM7 descriptors, delayed and incomplete resection, solid histologic subtype, and pleural relapse; had decreased OS and/or DFS (Figures 4A and 4F, Figures S5E and F, S6, S8C and D, S9B). All variables were entered into a second line least absolute shrinkage and selection operator (LASSO) regression analysis that identified age, body mass, percentage predicted FVC, FEV_1_/FVC ratio, percentage predicted DL_CO_, GOLD COPD stage, T, N, and cTNM7 stage, time to surgery, right lower lobe origin, and histologic subtype as determinants of OS and/or DFS (Figure 5A). In a final step, all variables that emerged both from Kaplan‐Meier and LASSO analyses were entered into Cox regression using backward Waldman elimination, which identified age outlying 45‐65 years, abnormal percentage predicted FVC and DL_CO_/V_A_, N2/3 disease, delayed resection, and solid histology as independent predictors of OS of GLAD (Figure 5B).

### 3.12 The locoregional lung adenocarcinoma death risk score (LADERS)

**Figure 6:**
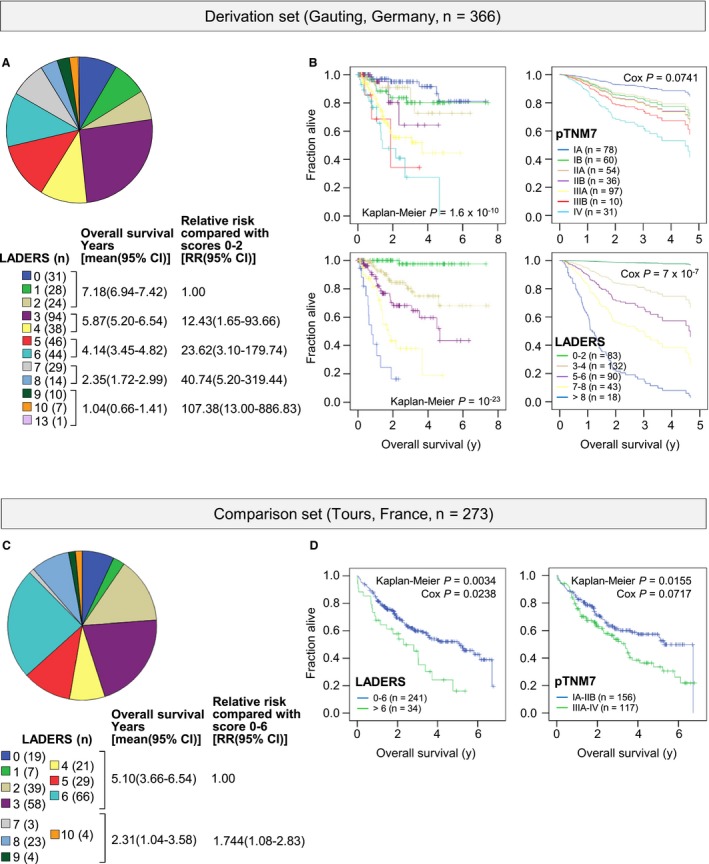
Performance of LADERS and pTNM7 as prognosticators in two locoregional lung adenocarcinoma patient cohorts. (A, B) Results from the GLAD derivation cohort. (C, D) Results from the Tours validation cohort. (A, C) Shown are LADERS distribution pie charts and patient numbers (*n*), LADERS groupings employed, and mean (because median was not reached for low LADERS scores) Kaplan‐Meier survival and Cox proportional hazards estimates with 95% confidence intervals (95%CI) of LADERS groupings. (B, D) Shown are Kaplan‐Meier and Cox proportional hazards survival plots and overall log‐rank test and Cox probability values (*P*) for LADERS and pTNM7 groupings, showing that LADERS more accurately predicted death events. TNM, tumor‐node‐metastasis staging system; p, pathologic; LADERS, locoregional lung adenocarcinoma death risk score

We next built a model to predict OS at the 30‐day postresection benchmark, using the six variables that withstood Kaplan‐Meier, LASSO, and Cox regression testing using OS as the target. LADERS employs Cox proportional hazard points and was tailored for easy clinical use without extra imaging/procedures (Figures 5C and 6A and B, Table 1). LADERS displayed only 25% correlation with pTNM7 and was intimately linked with death events, while pTNM7 showed tight linkage with relapse events (Figure S10A and B). LADERS outperformed pTNM7 in predicting OS of GLAD in Kaplan‐Meier and Cox regression analyses, while pTNM7 performed better in predicting DFS (Figure S10C; Table 2). LADERS also outperformed pTNM7 in predicting OS in the Tours cohort (Figure 6C and D).

**Table 1:** Independent predictors of survival identified by proportional hazards Cox regression analysis and locoregional lung adenocarcinoma death risk score (LADERS)

Variable	Hazard ratio (95% confidence interval)	Probability	Hazard points
Age <45 or >65 years	4.12 (2.35‐7.23)	0.0000008	3
FVC^a^ <80% predicted	2.13 (1.25‐3.63)	0.0054374	1
DL_CO_/V_A_ b <70% predicted	2.62 (1.60‐4.29)	0.0001292	2
N2^c^	3.56 (2.20‐5.76)	0.0000002	2.5
N3^c^	8.65 (1.10‐68.21)	0.0406576	7.5
Time to surgery >60 days^d^	4.04 (2.07‐7.88)	0.0000408	3
Solid histologic subtype^e^	2.09 (1.27‐3.43)	0.0035422	1
LADERS^f^			0‐20

a FVC, forced vital capacity. Compared with FVC ≥80% predicted. When FVC not available, GOLD COPD ≥stage II was used in the Tours cohort.

b DL_CO_/V_A_, Lung diffusion capacity for carbon monoxide corrected for alveolar ventilation. Compared with DL_CO_/V_A_ ≥70% predicted. When DL_CO_/V_A_ not available, current smoking was used in the Tours cohort.

c N, cTNM7 nodal status descriptors. Compared with pooled patients with N0 and N1.

d Compared with patients operated within 60 days from diagnosis.

e Compared with all other histologic subtypes combined, including lepidic, acinar, papillary, micropapillary, adenosquamous, fetal, and non‐specified.

f Rounded to the lower integer when decimal.

**Table 2:** Performance of pTNM7 and LADERS scores as predictors of survival of GLAD patients at discharge from thoracic surgery (30 days postsurgery census)

Score^a^	Outcome^b^	Cox regression^c^						Survival^d^		
		ND	DEV	DOF	AIC	CON	R^2^	iAUC	B‐score	B vs KM
pTNM7	OS	775.33	725.27	6	737.3	0.729	0.128	0.676	0.130	0.301
LADERS	OS	775.33	**671.58**	11	**693.6**	**0.804**	**0.247**	**0.764**	**0.118**	**0.366**
pTNM7	DFS	1727.57	**1569.58**	6	**1581.6**	**0.770**	**0.351**	**0.709**	**0.156**	**0.310**
LADERS	DFS	1727.57	1645.75	11	1667.8	0.701	0.200	0.672	0.182	0.195

The bold values highlight the outperformance of LADER or pTNM7 in the different tests.

a pTNM7, pathologic tumor‐node‐metastasis staging system, 7^th^ edition; LADERS, lung adenocarcinoma death risk score.

b OS, overall survival; DFS, disease‐free survival.

c Cox regression parameters: ND, null deviance; DEV, deviance; DOF, degrees of freedom; AIC, area in the curve; CON, concordance; R^2^, Pearson's correlation coefficient.

d Survival parameters: iAUC, integral area under the curve; B‐score, Brier’ concordance score; B vs KM, Brier skill vs Kaplan‐Meier.

## 4 DISCUSSION

Here we present GLAD, a prospectively evolving biobank of phenotypes and tumor/normal paired tissues of patients with locoregional LADC. The longitudinal follow‐up of the cohort suggests that locoregional LADC is currently a chronic lung disease with median survival >7.5 years. We corroborate pertinent findings of previous studies, such as the high frequency of these tumors in never/ex‐smokers and women and the significant overlap of LADC with COPD, the upper lobe predominance of these tumors that appears to be dexterous in smokers, as well as the value of current staging and histologic typing systems in management and prognosis.^1^, ^2^, ^22^ Using detailed phenotyping and prolonged follow‐up, we discovered previously ill‐defined and undefined clinical associations, such as the adverse effects of extreme age, poor lung function, and delayed resection on survival, as well as the early nature of pleural and the latency of pulmonary relapse. Most of our findings are corroborated in a separate patient cohort from France. Most importantly, we combined this wealth of clinical information to produce LADERS, a clinical score that accurately predicts survival in both cohorts.

In accord with only one previous report,^28^ young GLAD patients developed more aggressive LADC, possibly attributable to germline tumor suppressor loss, a hypothesis that can be directly tested in GLAD tissues. On the other hand, extremely old patients appeared to have a worse surgical prognosis associated with reduced lung and overall function. Active smokers had low N stage and relapse rates, findings possibly related with the young age and increased surveillance of active smokers in our cohort.

For the first time, we report how important time to surgery is for incipient survival, underscoring the aggressiveness of the disease and the urgency of surgery. We also define a previously reported spatial pattern of LADC development in the upper lobes.^29^ Although the clinical importance of this finding is unclear, it is likely the result of increased local conversion of inhaled precarcinogens to active carcinogens in the upper lobes of smokers. Of special note, we identify distinct temporal trends in organ‐specific relapse of early‐stage LADC, similar to biphasic metastatic patterns of other tumor types like breast cancer.^30^ Importantly, we provide clinicians with LADERS, an easy‐to‐use and accurate clinical tool to predict survival.

In conclusion, the first results from a prospective cohort of patients with locoregional LADC corroborate the impact of current staging and histologic subtyping systems and identify important effects of age, lung function, and time to resection on survival. A clinical tool to assess survival is also provided. Importantly, future combination of clinical information with tissue profiling is anticipated to unveil novel tumor genome‐phenome links and unprecedented mechanistic insights into evolution of carcinogenesis in the respiratory tract.

## CONFLICT OF INTEREST

The authors declare no conflict of interest.
