# Supplementary material for: Comprehensive clinical profiling of the Gauting locoregional lung adenocarcinoma donors
Source: Cancer Med. 2019 Feb 25;8(4):1486–99. doi: 10.1002/cam4.2031 (PMC6488114; doi:10.1002/cam4.2031)
Supplement: Supplementary file 1 [file CAM4-8-1486-s001.pdf]

A

n	Never smokers	Former smokers	Current smokers	Stage T1a	Stage T1b	Stage T2a	Stage T2b	Stage T3	Stage T4	Stage N0	Stage N1	Stage N2/N3	$DL_{CO}/V_A \geq 70\%$ predicted	$DL_{CO}/V_A < 70\%$ predicted	Time to surgery 0-60 days	Time to surgery > 60 days
Age < 45 years	2	1	8	2	0	3	2	2	2	1	3	7	7	4	7	4
Age 45-65 years	22	43	95	31	19	52	21	23	14	89	22	49	93	67	146	14
Age > 65 years	51	86	58	20	37	73	22	36	7	112	30	53	139	56	184	11
	$P = 3 \times 10^{-7}$			$P = 0.024$						$P = 0.035$			$P = 0.038$		$P = 0.006$	

					Tumor at post-surgery census				Postoperative relapse: Any		Postoperative relapse: Lung					
n	No death events	Death events	No relapse events	Relapse events	No	Yes	Not *EML4/ALK*-tested	*EML4/ALK*-tested	No	Yes	No	Yes	No lung relapse	Lung relapse	No bone relapse	Bone relapse
Age < 45 years	7	4	3	8	6	5	6	5	6	5	7	4	6	5	9	2
Age 45-65 years	141	19	100	60	136	24	137	23	119	41	133	27	121	39	158	2
Age > 65 years	144	51	99	96	159	36	169	56	121	74	138	57	126	69	191	4
	$P = 0.001$		$P = 0.014$		$P = 0.036$		$P = 0.030$		$P = 0.029$		$P = 0.010$		$P = 0.038$		$P = 0.020$	

Color key
0-20%
20-40%
40-60%
60-80%
80-100%

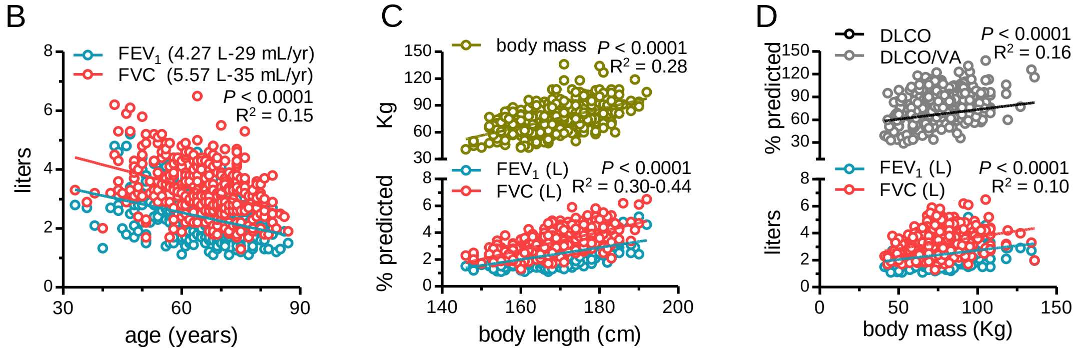

Figure S1. Impact of age on GLAD.

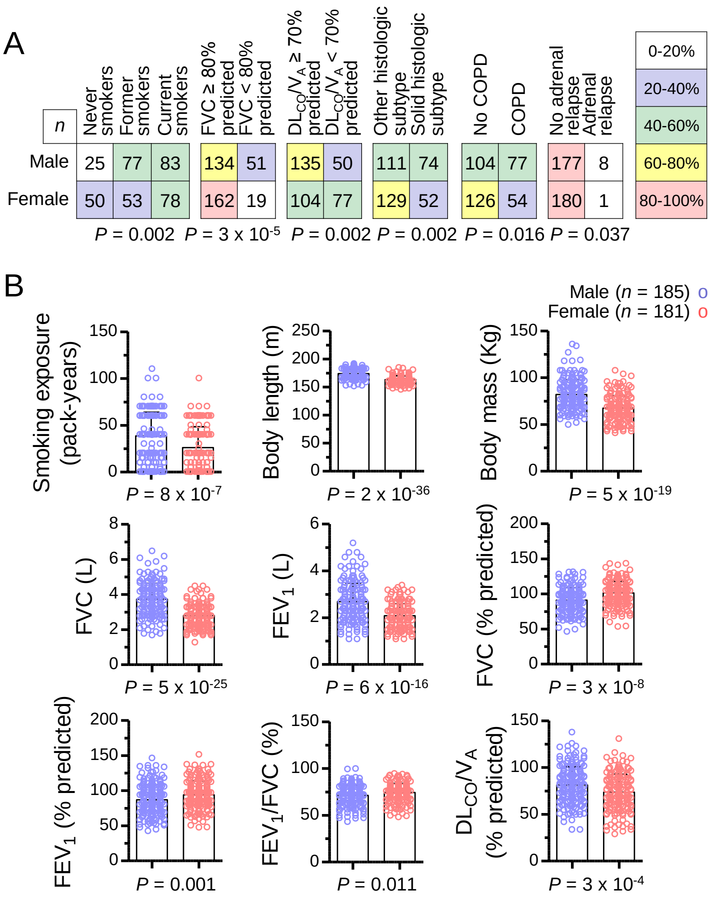

Figure S2. Associations with sex in the GLAD cohort.

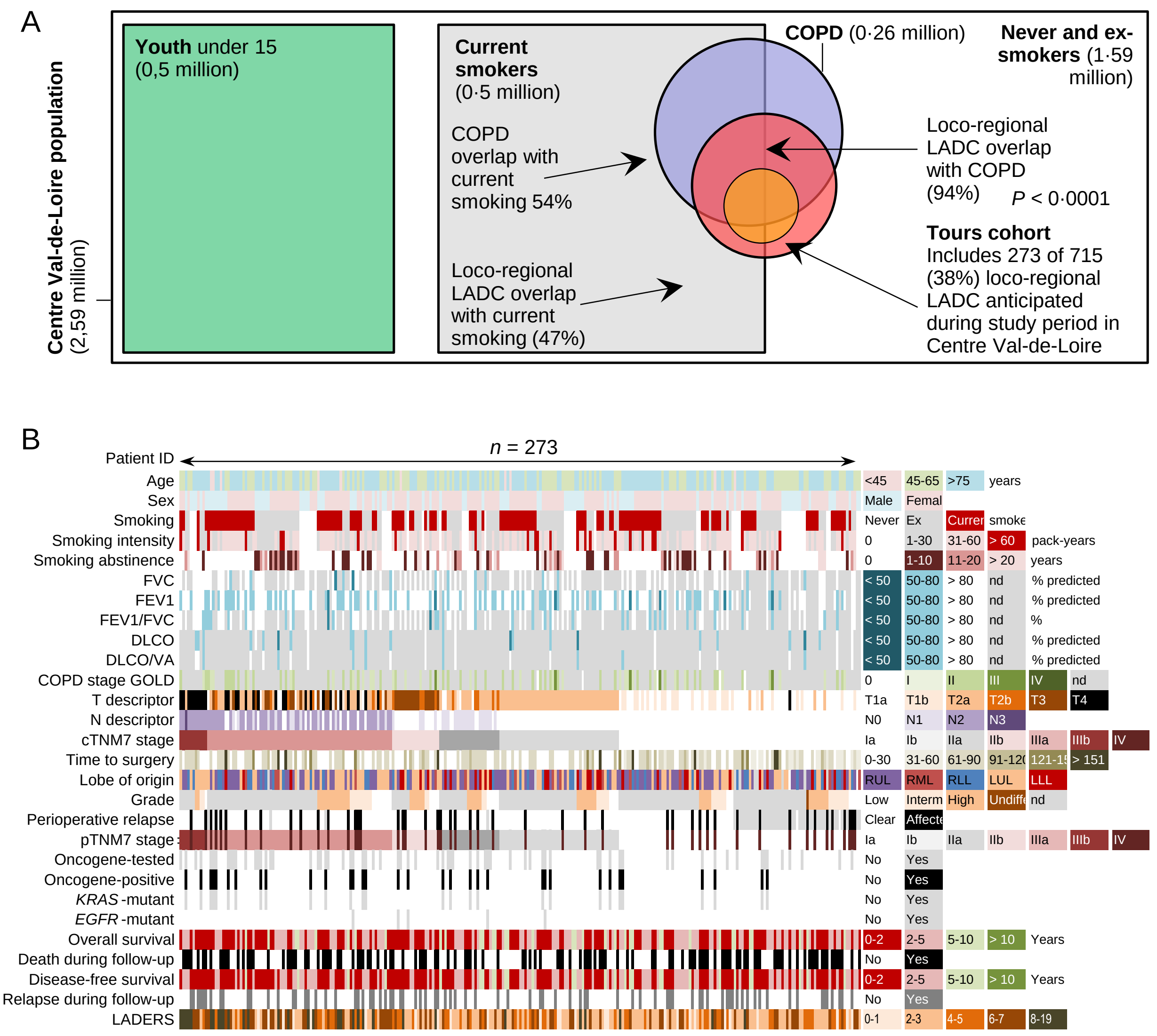

Figure S3. The Tours locoregional lung adenocarcinoma donors cohort.

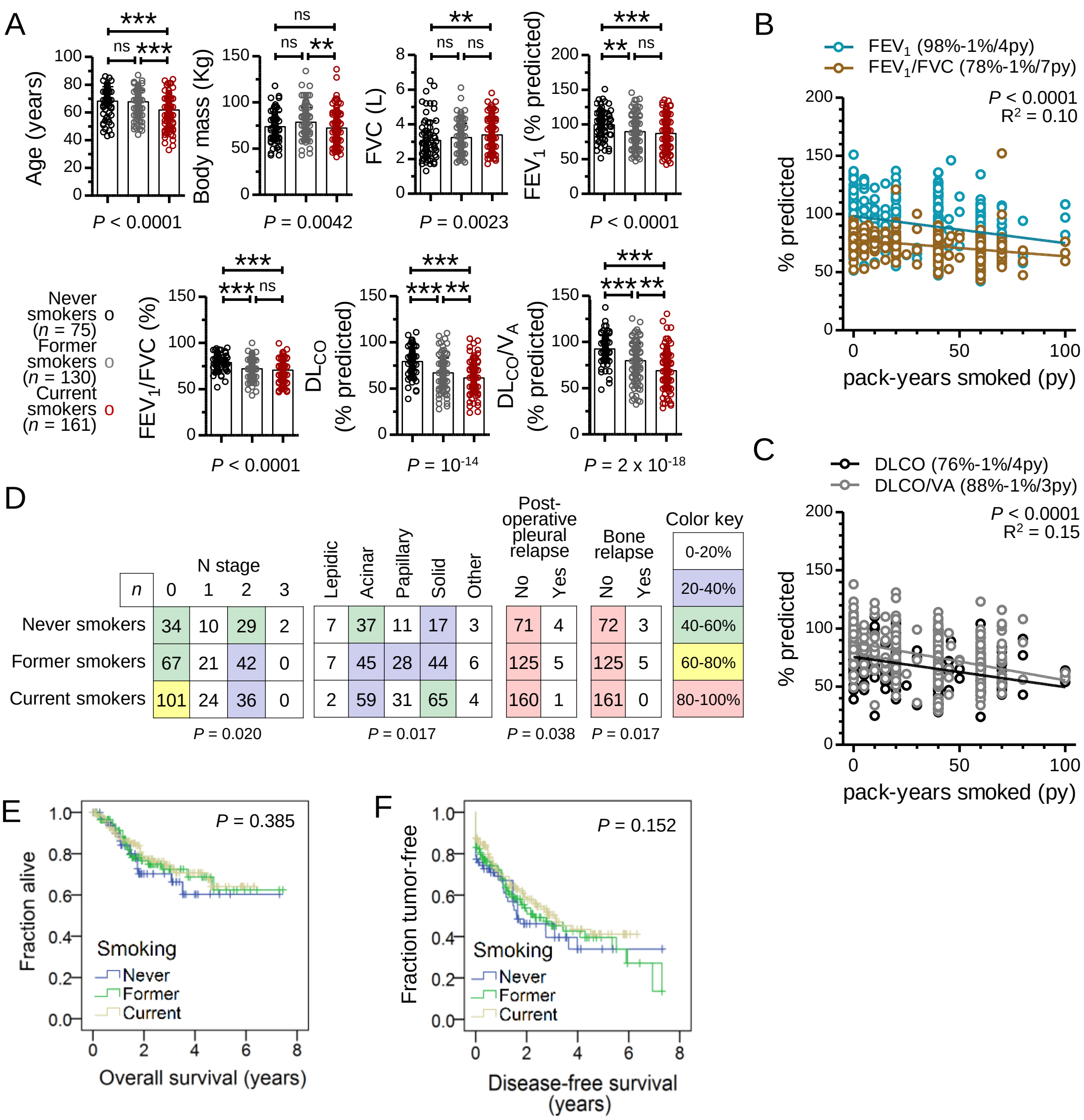

Figure S4. Smoking status, exposure, and effects in the GLAD cohort.

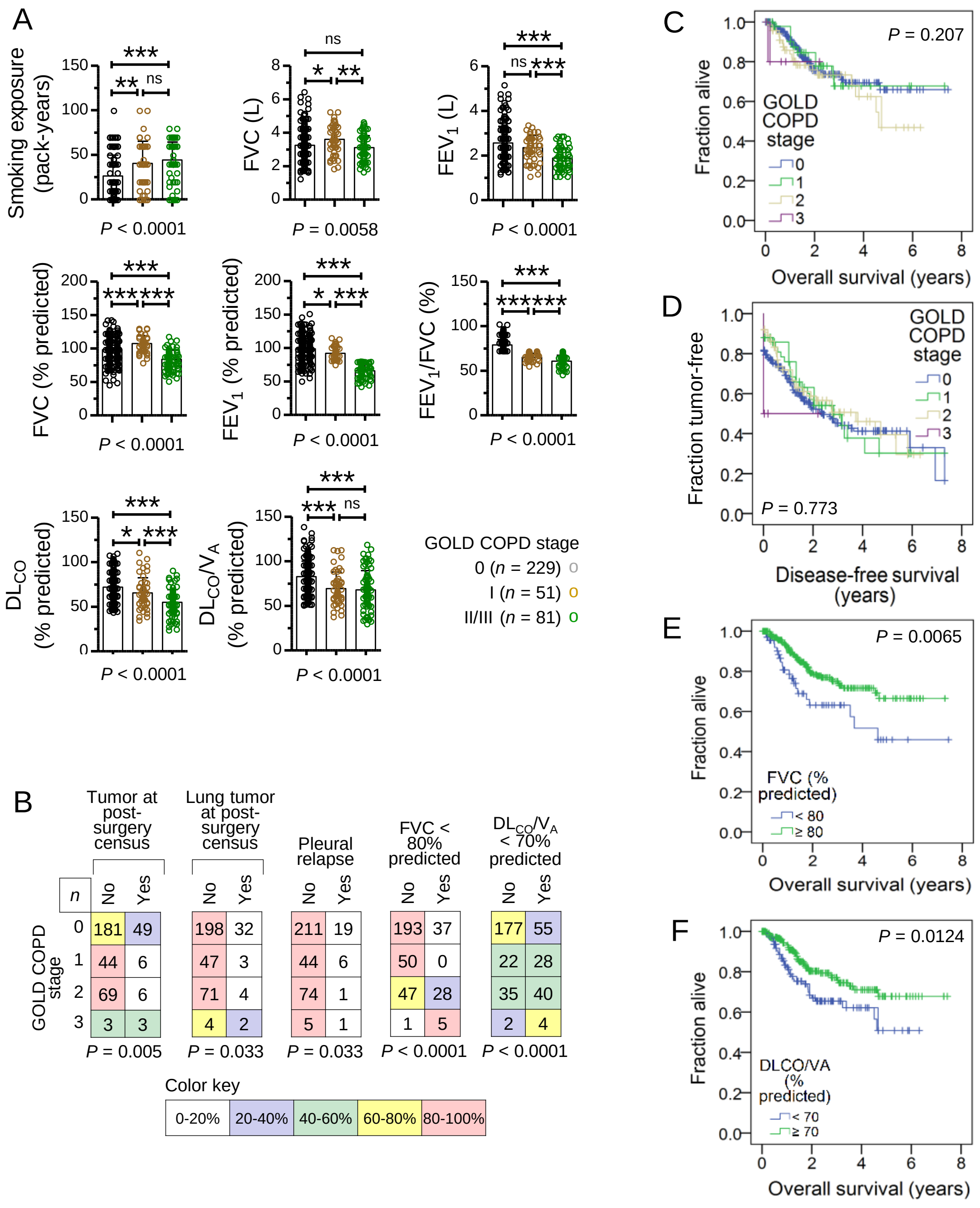

Figure S5. Chronic obstructive pulmonary disease (COPD) in the GLAD cohort.

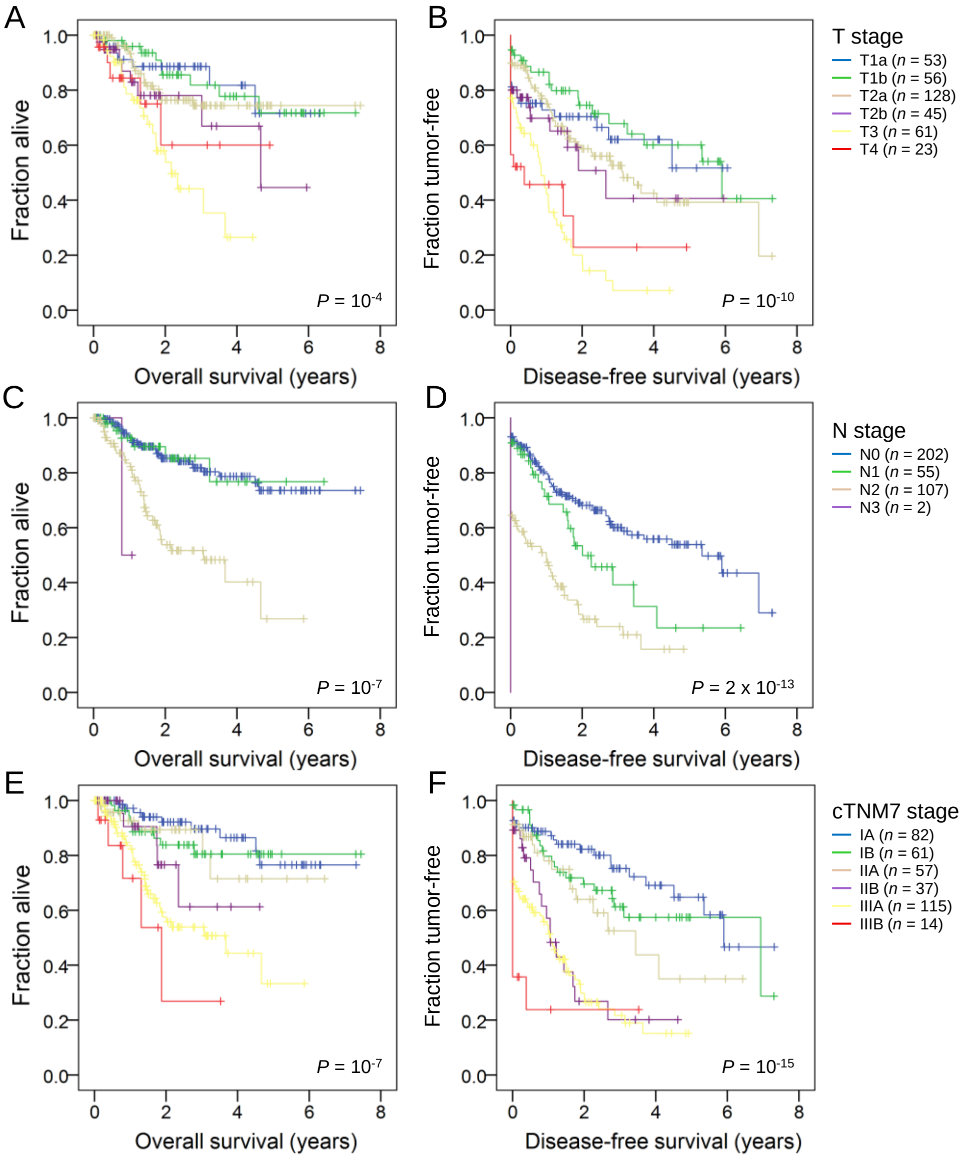

Figure S6. Validation of GLAD cTNM7 staging.

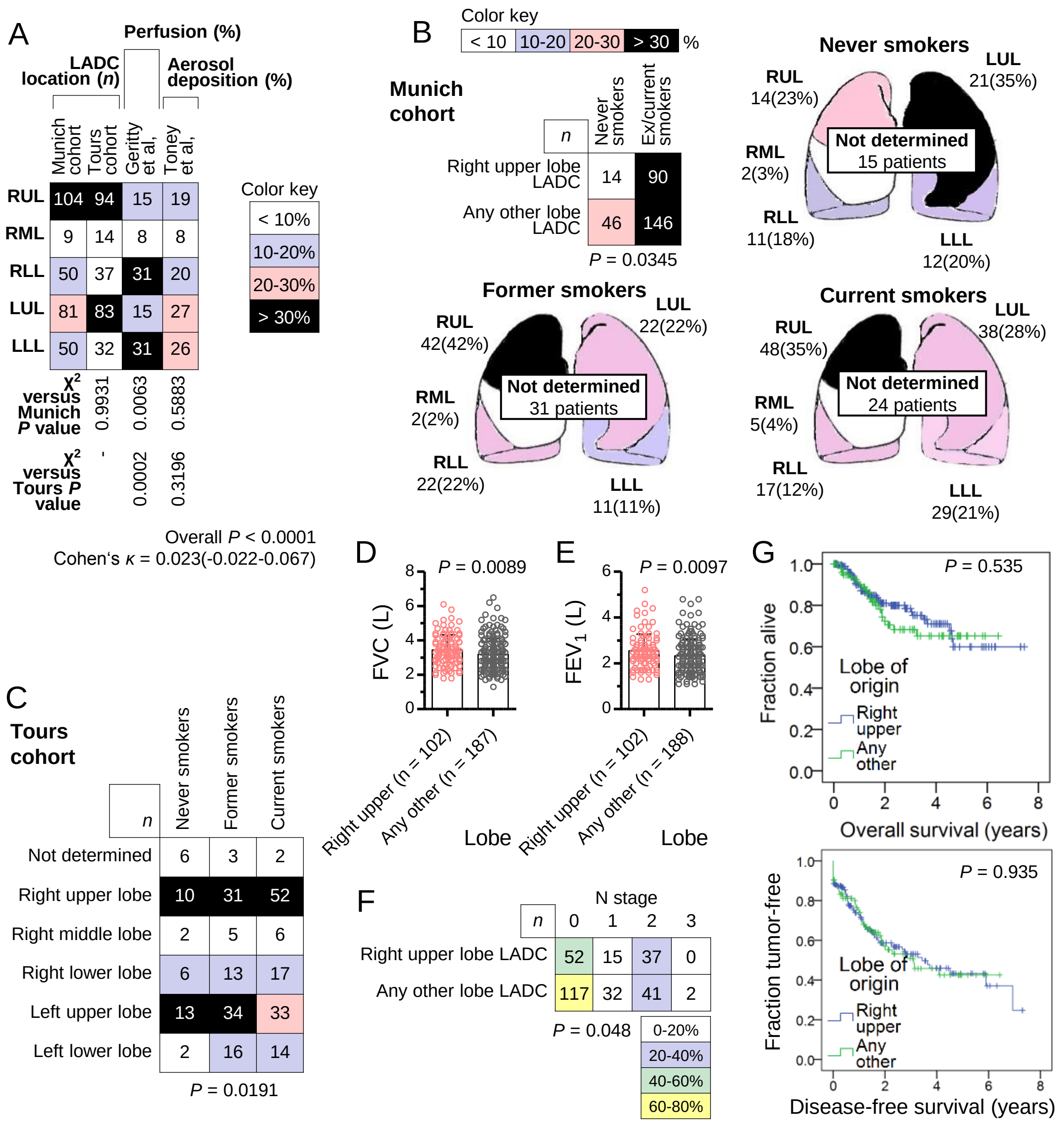

	Munich cohort	Tours cohort	Geritty et al,	Toney et al,
RUL	104	94	15	19
RML	9	14	8	8
RLL	50	37	31	20
LUL	81	83	15	27
LLL	50	32	31	26
χ^2 versus Munich P value		0.9931	0.0063	0.5883
χ^2 versus Tours P value		-	0.0002	0.3196

n	Never smokers	Ex/current smokers
Right upper lobe LADC	14	90
Any other lobe LADC	46	146

	Never smokers	Former smokers	Current smokers
RUL	14(23%)	42(42%)	48(35%)
RML	2(3%)	2(2%)	5(4%)
RLL	11(18%)	22(22%)	17(12%)
LUL	21(35%)	22(22%)	38(28%)
LLL	12(20%)	11(11%)	29(21%)

n	Never smokers	Former smokers	Current smokers
Not determined	6	3	2
Right upper lobe	10	31	52
Right middle lobe	2	5	6
Right lower lobe	6	13	17
Left upper lobe	13	34	33
Left lower lobe	2	16	14

P = 0.0191

n	0	1	2	3
Right upper lobe LADC	52	15	37	0
Any other lobe LADC	117	32	41	2

P = 0.048 (0-20%, 20-40%, 40-60%, 60-80%)

Figure S7. Tumor location on the GLAD and Tours cohorts.

A

	T stage			N stage			cTNM7 stage			Tumor at post-surgery census: Any		Tumor at post-surgery census: Lung	
n	T1	T2	T3/4	N0	N1	N2/3	I	II	III	No	Yes	No	Yes
Lepidic	8	2	6	14	2	0	8	7	1	16	0	16	0
Acinar	50	68	23	89	22	30	73	29	39	124	17	133	8
Papillary	18	33	19	40	3	27	28	14	28	54	16	58	12
Solid	32	66	28	56	26	44	33	41	52	100	26	108	18
	P = 0.028			P = 0.0002			P = 0.0002			P = 0.034		P = 0.016	

	pTNM7 stage				Postoperative relapse: Any		Postoperative relapse: Lung		Any relapse		Lung relapse		Observed deaths	
	I	II	III	≥	No	Yes	No	Yes	No	Yes	No	Yes	No	Yes
Lepidic	8	7	1	0	7	9	7	9	7	9	7	9	15	1
Acinar	71	28	33	9	109	32	117	24	95	46	112	29	120	21
Papillary	26	13	24	7	45	25	51	19	35	35	44	26	58	12
Solid	32	39	45	10	78	48	93	33	63	63	83	43	89	37
	P = 0.001				P = 0.006		P = 0.01		P = 0.01		P = 0.003		P = 0.01	

0-20%
20-40%
40-60%
60-80%
80-100%

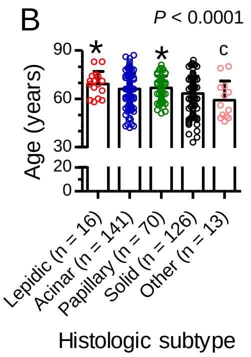

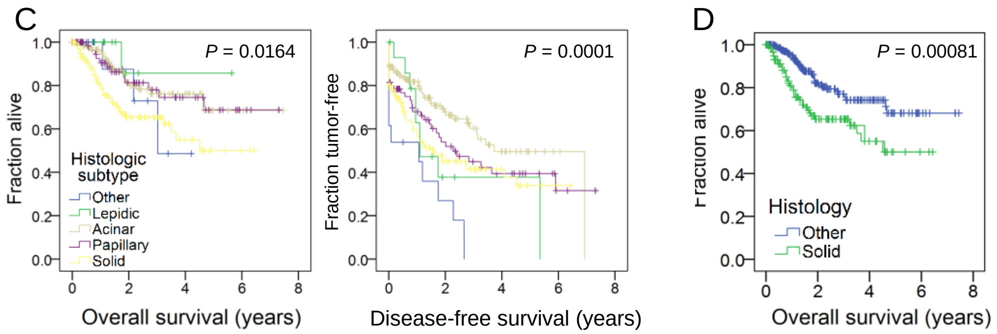

Figure S8. Impact of histologic subtype on outcomes in the GLAD cohort.

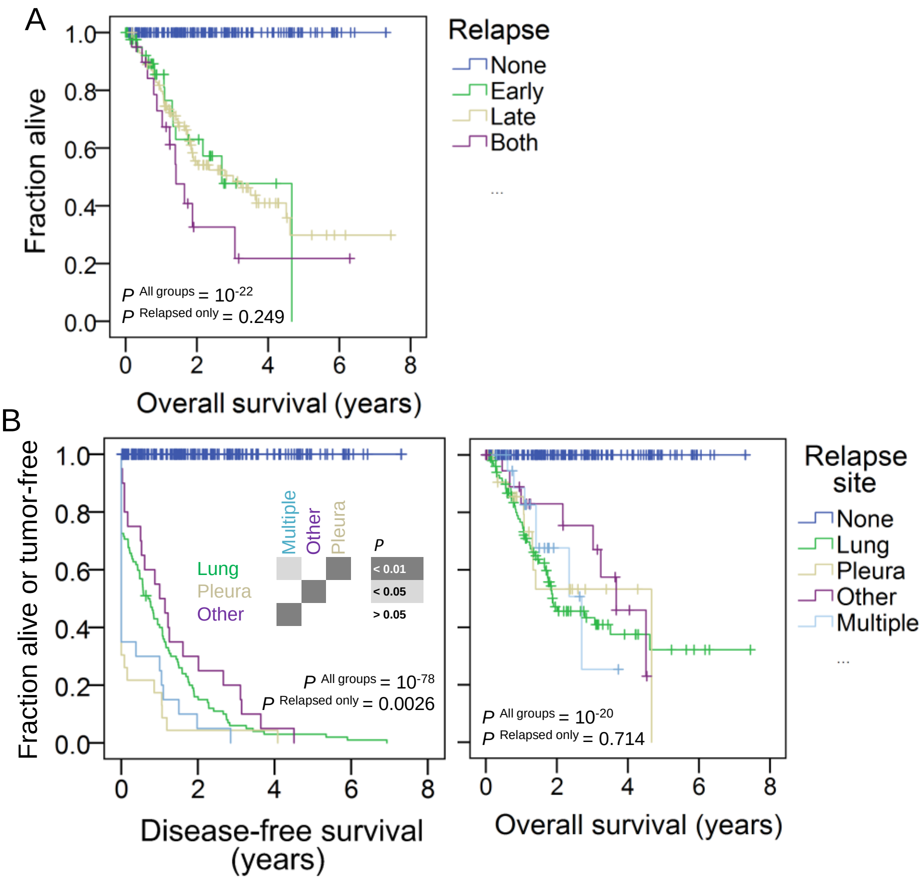

Figure S9. Patterns of relapse of GLAD..

A

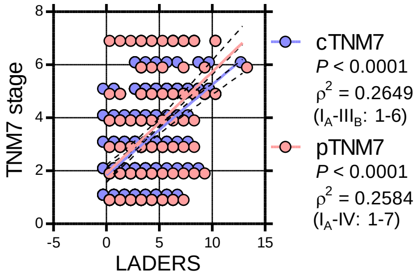

B

P	**Observed relapses**	**Observed deaths**
cTNM7	4×10^{-8}	6×10^{-6}
pTNM7	8×10^{-14}	9×10^{-7}
LADERS	10^{-6}	2×10^{-12}

C

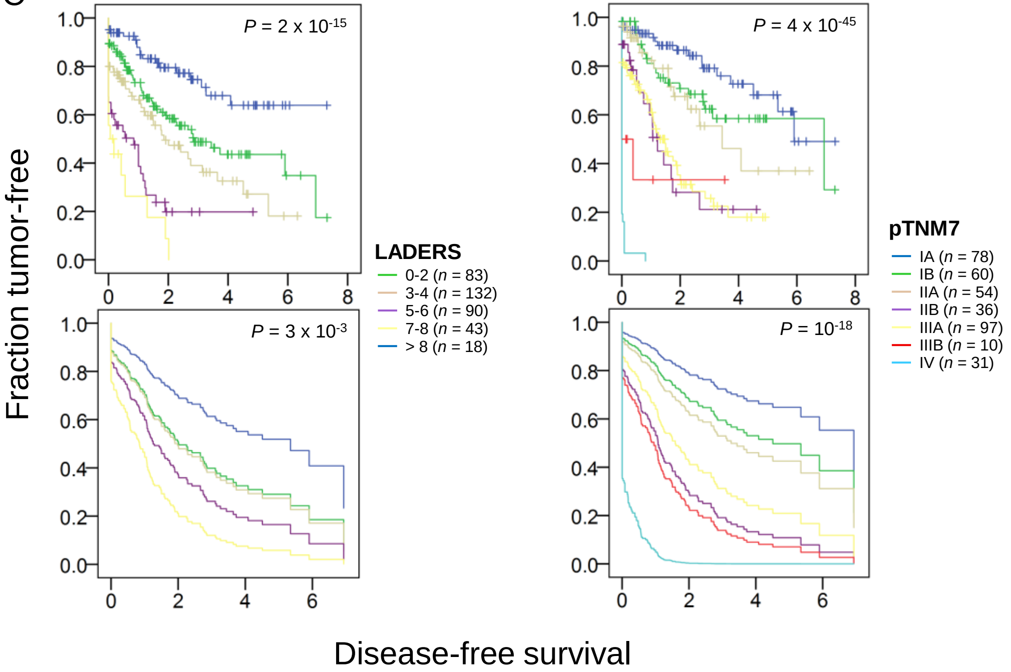

Figure S10. Comparison of the lung adenocarcinoma death risk score (LADERS) to TNM systems and their impact on disease-free survival.
