# Supplementary material for: Comprehensive clinical profiling of the Gauting locoregional lung adenocarcinoma donors
Source: Cancer Med. 2019 Feb 25;8(4):1486–99. doi: 10.1002/cam4.2031 (PMC6488114; doi:10.1002/cam4.2031)
Supplement: Supplementary file 4 [file CAM4-8-1486-s004.docx]

**SUPPLEMENTARY FIGURE LEGENDS**

**Comprehensive clinical profiling of the Gauting locoregional lung adenocarcinoma donors.**

**Figure S1.** **Impact of age on GLAD.** **(A)** Crosstabulations of GLAD by age groupings. Shown are patient numbers (*n*), color coded frequencies by age grouping, and Fischer’s exact probabilities (*P*). **(B-D)** Correlations of age and body metric indices with lung function tests (*n* = 366). Shown are raw data points (dots), linear regression lines and coefficients, and squared Pearson’s correlation coefficients (R^2^) and probabilities (*P*). *n*, sample size; DL_CO_, uncorrected lung diffusion capacity for carbon monoxide; V_A_, alveolar ventilation; *EML4*, echinoderm microtubule associated protein like 4; *ALK*, anaplastic lymphoma kinase; FVC, forced vital capacity; FEV_1_, forced expiratory volume in 1 sec.

**Figure S2. Associations with sex in the GLAD cohort**. **(A)** Crosstabulations of GLAD by sex. Shown are patient numbers (*n*), color coded frequencies by sex, and Fischer’s exact probabilities (*P*). **(B)** Smoking exposure, body metric indices, and lung function parameters stratified by sex. Shown are patient numbers (*n*), raw data points (dots), mean (columns), SD (bars), and Mann Whitney test probabilities (*P*).

**Figure S3. The Tours locoregional lung adenocarcinoma donors cohort**. **(A)** Venn diagram of current smoking COPD prevalence, and LADC incidence over the Tours study. **(B)** Phenome plot of the Tours cohort (raw data available in Table S2). Color-coded pivot table of all data obtained sorted sequentially by cTNM7 stage, tumor grade, sex, and smoking status. Columns represent individual patients and rows variables recorded. *n*, sample size; ID, identifier; FVC, forced vital capacity; FEV_1_, forced expiratory volume in 1 sec; DL_CO_, uncorrected lung diffusion capacity for carbon monoxide; V_A_, alveolar ventilation; COPD, chronic obstructive pulmonary disease; GOLD, global initiative for chronic obstructive lung disease; TNM, tumor-node-metastasis staging system; c, clinical; p, pathologic; *EGFR*, epidermal growth factor receptor; *KRAS,* V-Ki-ras2 Kirsten rat sarcoma viral oncogene homolog; LADERS, locoregional lung adenocarcinoma death risk score; nd, not determined.

**Figure S4. Smoking status, exposure, and effects in the GLAD cohort**. **(A)** Age, body mass, and lung function parameters stratified by smoking status. Shown are patient numbers (*n*), raw data points (dots), mean (columns), SD (bars), and Kruskal-Wallis test probabilities (*P*). ns, **, and ***: *P* > 0.05, *P* < 0.01, and *P* < 0.001, respectively, for the indicated comparisons by Dunn’s post-tests. **(B, C)** Correlations of smoking exposure with lung function tests (*n* = 366). Shown are raw data points (dots), linear regression lines and coefficients, and squared Pearson’s correlation coefficients (R^2^) and probabilities (*P*). **(D)** Crosstabulations of GLAD by smoking status. Shown are patient numbers (*n*), color coded frequencies by smoking status, and Fischer’s exact probabilities (*P*). **(E, F)** Kaplan-Meier disease-free and overall survival plots and overall log-rank test probability values (*P*) of the GLAD stratified by smoking status (*n* = 75, 130, and 161, respectively, for never, former, and current smokers). *n*, sample size; FVC, forced vital capacity; FEV_1_, forced expiratory volume in 1 sec; DL_CO_, uncorrected lung diffusion capacity for carbon monoxide; V_A_, alveolar ventilation.

**Figure S5. Chronic obstructive pulmonary disease (COPD) in the GLAD cohort**. **(A)** Age and lung function parameters stratified by COPD stage (*n* = 361 due to missing data in five patients) as defined by the global initiative for chronic obstructive lung disease 2001 criteria ^18^. Shown are patient numbers (*n*). raw data points (dots), mean (columns), SD (bars), and Kruskal-Wallis test probabilities (*P*). ns, *, **, and ***: *P* > 0.05, *P* < 0.05, *P* < 0.01, and *P* < 0.001, respectively, for the indicated comparisons by Dunn’s post-tests. **(B)** Crosstabulations of GLAD by GOLD COPD stage. Shown are patient numbers (*n*), color coded frequencies by age grouping, and χ^2^ probabilities (*P*). **(C, D)** Kaplan-Meier disease-free and overall survival plots and overall log-rank test probability values (*P*) of the GLAD stratified by GOLD COPD stage (*n* = 229, 51, 75, and 6, respectively, for GOLD COPD stages 0, I, II, and III). **(E, F)** Kaplan-Meier overall survival plots and log-rank test probability values (*P*) of the GLAD stratified by normal or abnormal forced vital capacity (FVC) and lung diffusion capacity for carbon monoxide corrected for alveolar ventilation (DL_CO_/V_A_) (FVC: *n* = 296 and 70, respectively, for values ≥ 80% and < 80%; DL_CO_/V_A_: *n* = 239 and 127, respectively, for values ≥ 70% and < 70%). *n*, sample size; FEV_1_, forced expiratory volume in 1 sec; DL_CO_, uncorrected lung diffusion capacity for carbon monoxide.

**Figure S6. Validation of GLAD cTNM7 staging**. Kaplan-Meier overall **(A, C, E)** and disease-free **(B, D, F)** survival plots and overall log-rank test probability values (*P*) of the GLAD stratified by T (A, B), N (C, D), and cTNM7 (E, F) stage. *n*, sample size; TNM, tumor-node-metastasis staging system; c, clinical.

**Figure S7. Tumor location in the GLAD and Tours cohorts**. **(A)** Crosstabulation of LADC location by lung lobe in the GLAD and Tours cohorts with lobar ventilation patterns determined by inhaled particle deposition and lobar perfusion patterns assessed via injected radioisotope distribution. Shown are number (*n*) of LADC observed, percentage of inhaled or injected particle distribution, overall χ^2^ probability (*P*) value, χ^2^ probability (*P*) values for comparison of each study to GLAD and Tours cohorts, and overall Cohen’s κ coefficient of agreement. Color indicates frequency. **(B, C)** LADC location by lung lobe determined at surgery in the GLAD derivation cohort and a smoking-optimal comparison cohort from Tours, France that is presented in detail in Supplementary Table 2 and Figure 3. Shown are schematic representations and crosstabulations of the lungs with their five lobes (RUL, right upper lobe; RML, right middle lobe; RLL, right lower lobe; LUL, left upper lobe; LLL, left lower lobe) and the number (*n*) and percentage of tumors observed in never, former, and current smokers, and Fischer’s exact (B) or χ^2^ (C) probabilities (*P*). Color indicates frequency. **(D, E)** Selected lung function parameters stratified by lobar tumor location in the GLAD cohort. Shown are patient numbers (*n*), raw data points (dots), mean (columns), SD (bars), and Student’s t test probabilities (*P*). **(F)** Crosstabulation of tumor location by N stage in GLAD. Shown are patient numbers (*n*), color coded frequencies by age grouping, and Fischer’s exact probability (*P*). **(G)** Kaplan-Meier disease-free and overall survival plots and log-rank test probability values (*P*) of GLAD patients stratified by tumor location in the right upper (*n* = 104) or any other (*n* = 29) lung lobe. *n*, sample size; LADC, lung adenocarcinoma; FVC, forced vital capacity; FEV_1_, forced expiratory volume in 1 sec.

**Figure S8. Impact of histologic subtype on outcomes in the GLAD cohort**. **(A)** Crosstabulations of histologic subtype by stage, relapse, and death related variables. Shown are patient numbers (*n*), color coded frequencies by histologic subtype, and Fischer’s exact probabilities (*P*). **(B)** Age stratified by histologic subtype. Shown are patient numbers (*n*), raw data points (dots), mean (columns), SD (bars), and Kruskal-Wallis test probability (*P*). *: *P* < 0.05 for comparison to other histologic subtype control (c) by Dunn’s post-tests. **(C)** Kaplan-Meier disease-free and overall survival plots and overall log-rank test probability values (*P*) of GLAD patients stratified by distinct histologic subtypes (*n* = 13, 16, 141, 70, and 126, respectively, for other, lepidic, acinar, papillary, and solid subtypes). **(D)** Kaplan-Meier overall survival plot and log-rank test probability value (*P*) of GLAD patients classified into solid (*n* = 126) and other non-solid (*n* = 240) histologic subtypes. *n*, sample size; TNM, tumor-node-metastasis staging system; c, clinical; p, pathologic.

**Figure S9. Patterns of relapse of GLAD. (A)** Kaplan-Meier overall survival plot and overall log-rank test probabilities inclusive (*P* ^All groups^) and non-inclusive (*P* ^Relapsed only^) of patients without relapse of GLAD patients stratified by timing of relapse: no relapse (*n* = 201), early relapse (prior to the 30-day post-resection census; *n* = 45), late relapse (thereafter; *n* = 100), or both (*n* = 20). **(B)** Kaplan-Meier disease-free and overall survival plots and overall and pairwise (table insert) log-rank test probabilities inclusive (*P* ^All groups^) and non-inclusive (*P* ^Relapsed only^) of patients without relapse of GLAD patients stratified by site of relapse: no relapse (*n* = 201), pulmonary relapse (*n* = 102), pleural relapse (*n* = 23), other extrathoracic relapse (*n* = 20), or multiple relapse sites (*n* = 20; five had also pleural relapse). *n*, sample size; TNM, tumor-node-metastasis staging system; c, clinical.

**Figure S10. Comparison of the lung adenocarcinoma death risk score (LADERS) to TNM systems and their impact on disease-free survival. (A)** Correlations of LADERS with cTNM7 (blue) and pTNM7 (red; *n* = 366 for both). Shown are raw data points (dots), linear regression lines with 95% confidence intervals, and squared Pearson’s correlation coefficients (R^2^) and probabilities (*P*). **(B)** χ^2^ probability (*P*) values for crosstabulations of cTNM7, pTNM7, and LADERS with observed relapses and deaths in the GLAD (shown in B) show pTNM7 to be closest linked with relapse, but LADERS with death events. **(C)** Kaplan-Meier (top) and Cox proportional hazards (bottom) disease-free survival plots and overall log-rank test and Cox probability values (*P*) for LADERS and pTNM7 groupings confirm the closer linkage of pTNM7 to relapse events. *n*, disease; TNM, tumor-node-metastasis staging system; c, clinical; p, pathologic.
