# Supplementary material for: Comprehensive clinical profiling of the Gauting locoregional lung adenocarcinoma donors
Source: Cancer Med. 2019 Feb 25;8(4):1486–99. doi: 10.1002/cam4.2031 (PMC6488114; doi:10.1002/cam4.2031)
Supplement: Supplementary file 5 [file CAM4-8-1486-s005.docx]

**SUPPLEMENTARY MATERIALS**

**Comprehensive clinical profiling of the Gauting locoregional lung adenocarcinoma donors.**

**Table S1** provided as a separate *.xlsx file.

**Table S2** provided as a separate *.xlsx file.

**Table S1 provided as a separate *.xlsx file.** Raw data obtained from the 366 Gauting locoregional lung adenocarcinoma donors (GLAD). ID, identifier; FVC, forced vital capacity; FEV_1_, forced expiratory volume in 1 sec; DL_CO_, uncorrected lung diffusion capacity for carbon monoxide; V_A_, alveolar ventilation; COPD, chronic obstructive pulmonary disease; GOLD, global initiative for chronic obstructive lung disease; TNM, tumor-node-metastasis staging system; c, clinical; p, pathologic; *EGFR*, epidermal growth factor receptor; *KRAS,* V-Ki-ras2 Kirsten rat sarcoma viral oncogene homolog; *BRAF,* v-Raf murine sarcoma viral oncogene homolog B; *EML4*, echinoderm microtubule-associated protein-like 4; *ALK*, anaplastic lymphoma kinase; LADERS, locoregional lung adenocarcinoma death risk score; nd, not determined.

**Table S2 provided as a separate *.xlsx file.** Raw data obtained from the 273 Tours locoregional lung adenocarcinoma donors. ID, identifier; FVC, forced vital capacity; FEV_1_, forced expiratory volume in 1 sec; DL_CO_, uncorrected lung diffusion capacity for carbon monoxide; V_A_, alveolar ventilation; COPD, chronic obstructive pulmonary disease; GOLD, global initiative for chronic obstructive lung disease; TNM, tumor-node-metastasis staging system; c, clinical; p, pathologic; *EGFR*, epidermal growth factor receptor; *KRAS,* V-Ki-ras2 Kirsten rat sarcoma viral oncogene homolog; LADERS, locoregional lung adenocarcinoma death risk score; nd, not determined.
